# Giant Cells of Various Lesions Are Characterised by Different Expression Patterns of HLA-Molecules and Molecules Involved in the Cell Cycle, Bone Metabolism, and Lineage Affiliation: An Immunohistochemical Study with a Review of the Literature

**DOI:** 10.3390/cancers15143702

**Published:** 2023-07-21

**Authors:** Vivien Hild, Kevin Mellert, Peter Möller, Thomas F. E. Barth

**Affiliations:** Institute of Pathology, University Hospital Ulm, 89081 Ulm, Germany

**Keywords:** giant cell, various lesions, immunohistochemical profiling, differential diagnosis

## Abstract

**Simple Summary:**

Giant cells (GCs) are found in many different tissues and among different backgrounds, including reactive, and neoplastic settings. It is not yet fully understood whether GCs in similar settings or with related functions share the same surface antigen expression. We performed antigen profiling of GCs via immunohistochemistry in multiple reactive and neoplastic lesions. We were able to characterise distinct groups of GCs with similar expression patterns, as well as GCs in some lesions that had a unique antigen expression pattern. These findings may help in the diagnosis of histologically similar GC-rich lesions and provide further insight into the function and origin of these cells.

**Abstract:**

Giant cells (GCs) are thought to originate from the fusion of monocytic lineage cells and arise amid multiple backgrounds. To compare GCs of different origins, we immunohistochemically characterised the GCs of reactive and neoplastic lesions (*n* = 47). We studied the expression of 15 molecules including HLA class II molecules those relevant to the cell cycle, bone metabolism and lineage affiliation. HLA-DR was detectable in the GCs of sarcoidosis, sarcoid-like lesions, tuberculosis, and foreign body granuloma. Cyclin D1 was expressed by the GCs of neoplastic lesions as well as the GCs of bony callus, fibroid epulis, and brown tumours. While cyclin E was detected in the GCs of all lesions, p16 and p21 showed a heterogeneous expression pattern. RANK was expressed by the GCs of all lesions except sarcoid-like lesions and xanthogranuloma. All GCs were RANK-L-negative, and the GCs of all lesions were osteoprotegerin-positive. Osteonectin was limited to the GCs of chondroblastoma. Osteopontin and TRAP were detected in the GCs of all lesions except xanthogranuloma. RUNX2 was heterogeneously expressed in the reactive and neoplastic cohort. The GCs of all lesions except foreign body granuloma expressed CD68, and all GCs were CD163- and langerin-negative. This profiling points to a functional diversity of GCs despite their similar morphology.

## 1. Introduction

Since the early description of multinucleated giant cells (GCs), these cells have been observed to emerge against different backgrounds [1,2]. Osteoclasts in healthy bone are known as physiological GCs. GCs associated with various pathological conditions may be further divided into GCs of reactive/inflammatory lesions like tuberculosis, sarcoidosis, foreign-body granuloma, and brown tumour, and GCs within neoplastic lesions. These include the so-called GC-containing lesions such as GC tumour of the bone, aneurysmal bone cysts, chondroblastoma, and giant-cell-containing osteosarcoma. Most GCs appear cytologically similar, with some differences in their cellular shape [3]. For instance, Langhans GCs in tuberculosis and sarcoidosis typically show a circular horseshoe-like pattern of nuclei, thereby differing from the GCs of other lesions, where the nuclei are arranged in the cytoplasm more randomly [4,5]. All GCs are polykaryons with a drastically varying number of nuclei, and most are derived from circulating inflammatory monocytes that, in turn, have developed from haematopoietic stem cells [3,6].

Throughout the decades, different theories of GC formation have been put forward. Two main paths of multinucleation have been suggested: acytokinetic cell division, and cell fusion [7]. In myxofibrosarcoma, mitotic activity and incomplete cytokinesis and re-fusion have been observed via time-lapse video microscopy [7]. Similar results have been produced for the formation of Reed-Sternberg cells from mononucleated Hodgkin cells in Hodgkin lymphoma [8]. However, this manner of formation has not yet been confirmed for other types of giant cells.

Today, a fusion of monocytes that have developed into macrophages is widely accepted as the main mechanism of formation for GCs [9]. This seems to be especially true for physiological GCs [7]. In vitro, monocytes and early macrophages have an enhanced ability to fuse and form GCs [10]. There are several different subtypes of macrophages derived from monocytes, which are defined by specific surface antigens, localisations, and capabilities [11]. GCs are thought to mainly originate from inflammation-resolving macrophages (M2a-d), although some are thought to be derived from inflammatory macrophages (M1) [3]. The process of GC formation seems to be much more complex than initially assumed and it is thought to reflect functional aspects of the GCs in each background. It has been suggested that multinucleation in neoplasms is the result of a weak contractile ring of the cytoskeleton caused by genetic defects [7]. Furthermore, GC formation seems to be enhanced in inflammatory conditions like sarcoidosis [12]. Firstly, tissue-specific cytokines (e.g., interleukin-4 or interleukin-13 in the case of foreign body granuloma) lead to the activation of monocytes and differentiation into macrophages [3,13]. After development into macrophages, cell fusion is initiated by another set of cytokines specific to the type of tissue or lesion. In foreign body granuloma, this process is mediated by E-cadherin and DC-STAMP, as well as the mannose receptor [3]. In xanthogranuloma, the fusion of monocytes is initiated by macrophage-colony stimulating factor, IFN-γ or interleukin-6. The resulting macrophages then fuse to form Touton giant cells in a process that is thought to be connected to the activation of toll-like receptors [3]. Although the initiating and inducing molecules vary between the respective tissues, the fusion process of GCs is thought to follow the same sequence: formation of fusion-competent cells, migration and adhesion, and cytoplasmic sharing [3].

So far, two distinct categories of GCs have been established: osteoclasts of the bone as well as osteoclast-like GCs of GC-rich bone lesions; and so-called macrophage polykaryons of extraosseous GC-containing lesions. These include Langhans GCs of tuberculosis and sarcoidosis, as well as foreign body GCs [3]. The common lineage shared by both osteoclasts (and osteoclast-like GCs) and macrophage polykaryons is reflected in the expression of the pan-macrophage marker CD68 and the leukocyte common antigen CD45 [14]. Likewise, both types of GCs are known to lose CD163-expression, while the preceding tissue macrophages are generally CD163-positive [14]. As the development pathways separate, osteoclasts and osteoclast-like GCs present a different set of expressed antigens by mostly losing their human leukocyte antigens (HLA)-DR- and CD14-expression, which are still present in monocytes [11,14]. Given the similar antigen profile of osteoclasts and osteoclast-like GCs, it is assumed that they are formed from the same precursors and through the same mechanisms [3,14]. Despite their similar cytological appearance, GCs are characterised by a variety of functions, but they all share the capability of phagocytosis [5]. An overview of the lesions included in this study, their respective type of GC and the function they entail in the lesion is given in Table 1. We set out to characterise GCs in different backgrounds by immunohistological profiling using a broad panel of antibodies detecting HLA class II molecules and those involved in cell cycle progression, bone metabolism, and lineage.

## 2. Materials and Methods

### 2.1. Tissue Samples

For this study, tissue samples were selected by systematic screening of the tissue bank of the Institute of Pathology Ulm University, Germany. Samples were provided as formalin-fixed and paraffin-embedded (FFPE) tissue sections. All analysed samples are listed in Table 2. In the reactive/inflammatory cohort they included four samples of bony callus, three of sarcoidosis, two lymph nodes with sarcoid-like lesions (sample 8 of a resection specimen from rectal cancer and sample 9 of a resection specimen from pancreatic adenocarcinoma), three samples of tuberculosis, three lesions with foreign body granuloma and two fibroid epulis. Additionally, two tissue samples of brown tumour were included that were obtained from two distinct lesions of the same patient (samples 18a and b).

The neoplastic cohort included samples of eleven GC tumours of the bone, four of which represent the core biopsies and resected tissues of the same case respectively (samples 24a and b as well as 25a and b), and two of which represent resected tissue of a primary tumour and a local recurrence in the same patient (samples 26a and b). Seven samples of aneurysmal bone cysts were included, among them one incision biopsy and resected tissue of the same tumour (samples 32a and b). We further included three chondroblastomas, three non-ossifying fibromas, three tenosynovial GC tumours and one xanthogranuloma.

All diagnostic procedures were performed in accordance with histological subtyping as defined by the WHO classification 2020 [24].

This study was approved by the Ulm University Ethics Committee (06/2017), which allowed the usage of archived human material. Appropriate clinical information was obtained from case notes, and patient material and samples were treated in a coded manner. All analyses were performed in accordance with the Declaration of Helsinki.

### 2.2. Immunohistochemistry

Immunohistochemistry was performed on giant cell-containing tissue sections (1.5 µm). Following deparaffination in xylol and rehydration in a graded alcohol series, antigen retrieval was performed to further unmask the antigen. To achieve this, the sections were either placed in target retrieval solution (TRS pH 6.1) or EDTA (pH 8 or 9) and heated in a steamer for 25 min. Alternatively, the sections were heated in citrate buffer (pH 6) for 25 min in a microwave or pressure cooker.

For the primary antibody reaction, sections were first marked with a PAP pen and pre-incubated with phosphate-buffered saline (PBS), then covered with 50–100 µL primary antibody in the specified dilution and incubated in a humidified chamber for 30 min. The utilised primary antibodies are listed in Table 3.

**Figure 1:**
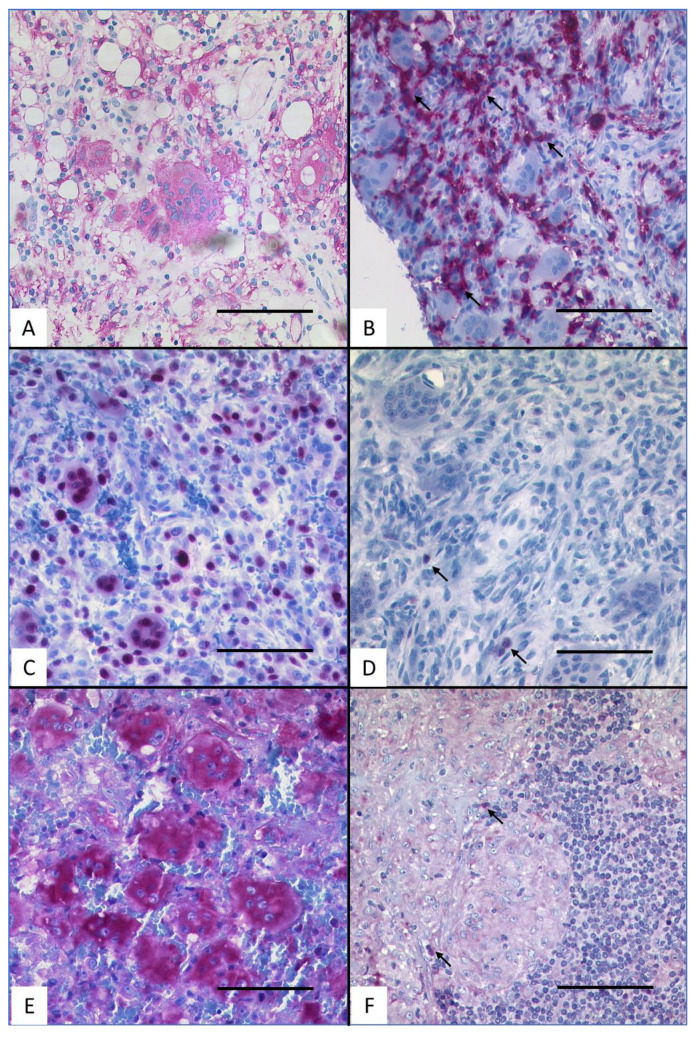
Typical immunohistochemistry for HLA-DR, p21 and RANK. (**A**) HLA-DR-positive staining in the GCs of tuberculosis. (**B**) HLA-DR-negative staining in the GCs of bony callus. (**C**) p21-positive staining in the GCs of fibroid epulis. (**D**) p21-negative staining in the GCs of bony callus. (**E**) RANK-positive staining in the GCs of GC tumour of the bone. (**F**) RANK-negative staining in the GCs of sarcoidosis (scale bar = 100 µm). Arrows indicate intrinsic positive control within negative staining examples.

For the secondary antibody reaction, Dako REAL Detection System Alkaline Phosphatase/RED rabbit/mouse (cat# K5005) was used. Counterstaining followed, using Meyer’s hematoxylin with an incubation time of 5 min. Evaluation was performed in a blinded, neutral manner and by consensus diagnosis using a multi—head microscope. Immunoreactivity of giant cells was categorised into four categories, depending on the proportion of positively stained cells: “no immunoreactivity detected”, “immunoreactivity in up to 30%”, “immunoreactivity in more than 30% and up to 70%” and “immunoreactivity in more than 70%” of the total number of analysed cells. A typical example of categorisation is given in Figure 1. Additional images of positive staining of different lesions and antigens can be found in the Supplementary Material Figures S1–S5. The bar marks the respective magnification.

## 3. Results

The pattern of expression of all studied antigens is shown in Table 4.

### 3.1. HLA Class II

#### 3.1.1. HLA-DR

##### Reactive Lesions

HLA-DR was positive in the GCs of sarcoidosis, sarcoid-like lesion, and foreign body granuloma. The GCs of tuberculosis stained positive for HLA-DR in up to 70%. The GCs of bony callus, fibroid epulis, and brown tumour were HLA-DR-negative.

##### Neoplastic Lesions

All GCs within neoplastic lesions were HLA-DR-negative.

### 3.2. Cell Cycle Group

#### 3.2.1. Cyclin D1

##### Reactive Lesions

Cyclin D1 was positive in the GCs of bony callus, fibroid epulis and brown tumour, while the GCs of foreign body granuloma were cyclin D1 positive in about 70% of the GCs. The GCs of sarcoidosis, sarcoid-like lesion, and tuberculosis were consistently cyclin D1-negative.

##### Neoplastic Lesions

Cyclin D1 was positive in the nuclei of the GCs of the studied neoplastic lesions throughout.

#### 3.2.2. Cyclin E

##### Reactive Lesions

Cyclin E was found to be expressed in the GCs of all lesions, although in varying percentages. In sarcoidosis, tuberculosis, and in one brown tumour, the percentage of stained GCs was up to 70%, while it was above 70% in all other studied reactive lesions. In the GCs of two samples of foreign body granuloma, the staining pattern of cyclin E was strictly cytoplasmatic, while it was nuclear restricted in one foreign body granuloma and in all other studied lesions.

##### Neoplastic Lesions

The GCs of all studied neoplastic lesions showed nuclear expression of cyclin E. More than 70% of GCs were positive in GC tumour of the bone, aneurysmal bone cyst, chondroblastoma, and non-ossifying fibroma. In tenosynovial GC tumour, up to 70% of the GCs were positive, and the GCs of xanthogranuloma showed up to 30% immunoreactivity.

#### 3.2.3. p16

##### Reactive Lesions

p16 was positive in over 70% of the GCs of fibroid epulis, over 30% of the GCs of foreign body granuloma, and in up to 30% of the GCs in bony callus. The GCs of sarcoidosis, sarcoid-like lesion, tuberculosis, and brown tumour were p16-negative.

##### Neoplastic Lesions

The GCs of aneurysmal bone cyst and tenosynovial GC tumour were positive for p16 in up to 70%, while the GCs of chondroblastoma were up to 30% positive.

In one sample of GC tumour of the bone, p16 was detected in the incision biopsy but not in the resected tissue (samples 25a and b). In another case, p16 was positive in up to 70% of GCs in the primary occurrence of the tumour, while the recurrence only showed positivity in up to 30% of GCs (samples 26a and b). In the GC tumours of the bone and aneurysmal bone cysts, the reaction was cytoplasmatic in some analysed samples, and strictly nuclear in others, pointing to a mutually exclusive expression of p16. The GCs of non-ossifying fibroma and xanthogranuloma were p16-negative.

#### 3.2.4. p21

##### Reactive Lesions

p21 was positive in bony callus and sarcoidosis in up to 30% of GCs, in up to 70% of GCs in tuberculosis, and the mandibular brown tumour, and in more than 70% of the GCs of foreign body granuloma, fibroid epulis and the brown tumour of the femur. The GCs of sarcoid-like lesion were p21-negative.

##### Neoplastic Lesions

Many of the neoplastic lesions were partially positive in GCs between 30% and 70%, namely GC tumour of the bone, aneurysmal bone cyst, chondroblastoma, non-ossifying fibroma, and tenosynovial GC tumour. In one sample of GC tumour of the bone, the GCs of the core biopsy and resected tissue showed a reaction in above 70% (24a and b), while results were incongruent in another sample of GC tumour of the bone (25a and b) and a sample of aneurysmal bone cyst (32a and b). The GCs of one sample stained partially positive, while the other remained negative, respectively. The GCs of xanthogranuloma were p21-negative.

### 3.3. Bone Metabolism Group

#### 3.3.1. Receptor Activator of Nuclear Factor κB (RANK)

##### Reactive Lesions

More than 70% of GCs showed immunoreactivity for RANK in bony callus, foreign body granuloma, fibroid epulis, and brown tumour. The GCs of sarcoidosis and tuberculosis were positive in 30–70%, while the GCs of sarcoid-like lesion were negative.

##### Neoplastic Lesions

The GCs of almost all neoplastic lesions were positive for RANK in more than 70%, namely the GCs of GC tumour of the bone, aneurysmal bone cyst, chondroblastoma, non-ossifying fibroma, and tenosynovial GC tumour. The GCs in xanthogranuloma showed no immunoreactivity for RANK.

#### 3.3.2. Receptor Activator of Nuclear Factor κB Ligand (RANK-L)

##### Reactive Lesions

Up to 30% RANK-L-positive GCs were found in bony callus, fibroid epulis, and in one of two brown tumours studied. The GCs of sarcoidosis, sarcoid-like-lesion, tuberculosis, foreign body granuloma, fibroid epulis, and one localisation of brown tumour were RANK-L-negative.

##### Neoplastic Lesions

The GCs of aneurysmal bone cyst, chondroblastoma, and tenosynovial GC tumour showed immunoreactivity for RANK-L in up to 30%. The GCs of GC tumour of the bone, non-ossifying fibroma, and xanthogranuloma were RANK-L-negative throughout.

#### 3.3.3. Osteoprotegerin (OPG)

##### Reactive Lesions

OPG-expression was detected in the GCs of all reactive lesions at a varying percentage. The GCs of bony callus, sarcoid-like lesion, tuberculosis, fibroid epulis, and one brown tumour expressed OPG in more than 70% of GCs, while sarcoidosis, foreign body granuloma and one brown tumour showed OPG-positivity in 30–70% of GCs.

##### Neoplastic Lesions

The GCs of all studied neoplastic lesions were more than 70% OPG-positive throughout.

#### 3.3.4. Osteonectin

##### Reactive Lesions

The GCs of almost all studied reactive lesions were osteonectin-negative throughout, namely bony callus, sarcoidosis, sarcoid-like lesion, tuberculosis, foreign body granuloma, fibroid epulis, and brown tumour.

##### Neoplastic Lesions

The GCs of neoplastic lesions were mostly negative throughout, including the GCs of GC tumour of the bone, non-ossifying fibroma, tenosynovial GC tumour, and xanthogranuloma. The GCs of aneurysmal bone cyst were immunoreactive in up to 30% in most studied samples. In one case, the GCs of the incisional biopsy of aneurysmatic bone cyst (sample 32a) were positive in up to 70% of cells, while the GCs of the tissue resected shortly thereafter (sample 32b) were osteonectin-negative.

#### 3.3.5. Osteopontin

##### Reactive Lesions

In the GCs of all studied reactive lesions, osteopontin was present at different levels. In bony callus, sarcoid-like lesion, tuberculosis, foreign body granuloma and one brown tumour, osteopontin was expressed in over 70% of GCs. In sarcoidosis and one brown tumour, osteopontin was present in 30–70% of the studied GCs.

##### Neoplastic Lesions

The GCs of GC tumour of the bone and tenosynovial GC tumour expressed osteopontin in over 70%, while chrondroblastoma and non-ossifying fibroma showed up to 70% osteopontin-positive GCs. In one chondroblastoma, the reaction observed was strictly cytoplasmatic, while it was strictly nuclear in the other two chondroblastomas. Most aneurysmal bone cysts were up to 70% osteopontin-positive in the GC compartment. However, sample 32a (incision biopsy) was osteopontin-negative, while sample 32b (resected tissue of the same lesion) was over 70% osteopontin-positive. The GCs of xanthogranuloma were osteopontin-negative.

#### 3.3.6. Tartrate Resistant Acid Phosphatase (TRAP)

##### Reactive Lesions

TRAP was expressed in the GCs of all reactive lesions to different degrees. In bony callus, sarcoid like lesion, foreign body granuloma and fibroid epulis, more than 70% of the analysed GCs were TRAP-positive. In sarcoidosis, tuberculosis and brown tumour, between 30% and 70% of GCs were TRAP-positive.

##### Neoplastic Lesions

All analysed neoplastic lesions expressed TRAP in the GC compartment. In GC tumour of the bone, aneurysmal bone cyst, chondroblastoma, non-ossifying fibroma, and tenosynovial GC tumour, the GCs were TRAP-positive throughout. The GCs of xanthogranuloma were TRAP-positive in up to 30%.

#### 3.3.7. Runt-Related Transcription Factor 2 (RUNX2)

##### Reactive Lesions

The GCs of sarcoid-like lesion, foreign body granuloma and fibroid epulis were above 70% RUNX2-positive, while the GCs of bony callus and sarcoidosis were between 30% and 70% RUNX2-positive. In tuberculosis, up to 30% of GCs were RUNX2-immunoreactive, and the GCs of brown tumour were RUNX2-negative throughout.

##### Neoplastic Lesions

The studied neoplastic lesions show a heterogenous profile, with lesions like GC tumour of the bone revealing different patterns within one group. Six samples of GC tumour of the bone (samples 19–23, and 25 a and b) reveal between 30% and 70% RUNX2-positive GCs. Sample 24a (core biopsy) shows 30–70% immunoreactive GCs, and the resected tissue 24b is above 70% RUNX2-positive in the GC compartment. Sample 26 is similar, with the biopsy showing up to 30% immunoreactivity, and the GCs of the resected tissue being RUNX2-positive throughout. The GCs of aneurysmal bone cyst were up to 30% RUNX2-positive, with the exception of sample 32a (incision biopsy), which was RUNX2-negative.

The GCs of tenosynovial GC tumour were RUNX2-positive throughout, while the GCs of non-ossifying fibroma were up to 30% RUNX2-positive, and the GCs of chondroblastoma and xanthogranuloma did not show immunoreactivity for RUNX2.

### 3.4. Differentiation Group

#### 3.4.1. CD68

##### Reactive Lesions

Almost all reactive lesions showed a high percentage (>70%) of CD68-positive GCs, one exception was GC of foreign body granuloma, with up to 30% positive GCs.

##### Neoplastic Lesions

All neoplastic lesions showed a varying percentage of reactive CD68-positive GCs. 30–70% of the GCs of chondroblastoma stained CD68-positive, while the GCs of all other lesions stained positive above 70%.

#### 3.4.2. CD163

##### Reactive and Neoplastic Lesions

None of the GCs in the studied reactive and neoplastic lesions showed immunoreactivity for CD163.

#### 3.4.3. Langerin

##### Reactive and Neoplastic Lesions

None of the GCs in the studied reactive and neoplastic lesions showed immunoreactivity for Langerin.

## 4. Discussion

We performed a comprehensive immunohistochemical analysis of the GCs of various reactive and neoplastic lesions and detected characteristic profiles of GCs in the lesions analysed. An overview of the tested molecules and their function is given in Table 5.

HLA-DR- one of the major histocompatibility complex class II molecules- is a surface molecule that presents exogenously derived antigens to CD4⁺ T-lymphocytes, thereby alerting the immune system to pathogens like viruses or bacteria [25]. Subsequently, these T-cells orchestrate macrophage activation and B- and T-cell differentiation and proliferation, which results in the production of pathogen-specific antibodies [25]. HLA-DR is typically expressed on antigen-presenting cells like B-cells, dendritic cells, and macrophages. These cells internalise foreign antigens that are then processed into fragments and presented on the cell surface [40]. In inflammatory conditions, a host of other cell types like vascular endothelial cells and dermal fibroblasts also show HLA-DR-expression and therefore antigen-presenting capabilities [41]. The GCs of bony callus and brown tumour have been shown to be HLA-DR-negative, thereby resembling osteoclasts and other osteoclast-like GCs [14]. Meanwhile, Langhans-GCs of tuberculosis and sarcoidosis, and foreign body GCs have been reported to be HLA-DR-positive like the macrophages they are derived from [14]. To the best of our knowledge, for the GCs of sarcoid like lesion and fibroid epulis, there are no data regarding HLA-DR-expression, and therefore our data add new information on GCs of different backgrounds regarding this antigen. As our data broadens the scope of antigen testing for many lesions analysed, our own new data which have been added are shortly referred to as “no published data” in the comments on this list. Although the GCs of fibroid epulis are likely to be macrophage polykaryons, they lacked expression of HLA-DR, while the GCs of sarcoid-like lesion showed the same expression as Langhans cells and stained positive for HLA-DR. The GCs in giant cell tumour of the bone (ICD-O 9250/1), chondroblastoma (ICD-O 9230/0), non-ossifying fibroma (ICD-O 8830/0) and aneurysmal bone cyst (ICD-O 9260/0) have been shown to lack HLA-DR-expression, in contrast to GC derived from macrophages in an antigen presenting setting; therefore, these cells seem to be similar to osteoclasts [14]. The CGs of tenosynovial GC tumour (ICD-O 9252/0, ex.: giant cell tumour of tendon sheath) have been shown to be HLA-DR-negative before [42], as have the GCs in xanthogranuloma [43]. Although these GCs are more similar to macrophage polykaryons than to osteoclasts, with these lesions at least two types of GC seem to exist in a low-grade inflammatory background with no antigen-presenting capabilities.

Cyclin D1 plays a key role in cell cycle progression from G1- to S-phase. It acts as a regulatory subunit of cyclin-dependent kinases (CDK) 4 and 6, that in turn phosphorylate the retinoblastoma protein, allowing the cell cycle to progress to the phase of DNA replication [26]. The GCs of bony callus have been shown to be cyclin D1-negative [44]. In contrast to these published data, we show that the GCs of bony callus are strongly cyclin D1-positive. To the best of our knowledge, there are no published data concerning the cyclin D1 expression of GCs in sarcoidosis, sarcoid-like lesion, tuberculosis, foreign body granuloma, and brown tumour. Cyclin D1-expression is induced/deregulated in many malignant tumours due to a gene dosage effect by amplification of the cyclin D1-gene (CCND1), which is one of the most frequently amplified gene loci in solid cancers [45]. Although no GCs were studied in these previous examples, we show that cyclin D1-expression in GC in our study was limited to the GCs of neoplastic lesions and was strong and consistent throughout. Namely, the GCs of aneurysmal bone cyst, chondroblastoma, non-ossifying fibroma, and tenosynovial GC tumour stained positive for this antigen. Cyclin D1 has been proved to be upregulated in the GCs of GC tumour of the bone [46]. This led to the assumption of a cell cycle in GCs, at least in the G1-phase [47]. Cyclin D1 might also play a role in GC formation and multinucleation, as opposed to purely cell proliferation in this tumour, as it has mostly been detected in smaller GCs with less nuclei [48]. It has been assumed that cyclin D1 may also be involved in tumorigenesis as a target of the Wnt pathway [44]. Additionally, nuclear cyclin D1 expression in GCs has been identified as an independent risk factor of GC tumour of the bone-recurrence after surgical therapy, as tumours displaying this trait have been known to be two times more recurrent than cyclin D1-negative tumours [49]. Immunostaining for cyclin D1 has been performed on xanthogranuloma before, confirming our results with positivity of cyclin D1 in the GCs [23]. This may point to at least one aberrant pathway in the GCs of xanthogranuloma, possibly a part of the RTK/MAPK-pathway [23].

Cyclin E, a common term used for cyclins E1 and E2, is another key player in cell cycle regulation: it binds protein kinase CDK2 and allows the cell to progress from G1- to S-phase by contributing to the phosphorylation of the retinoblastoma protein [50]. Consequently, cyclin E-levels peak during this specific phase transition [27]. Although the presence of cyclin E seems to be essential in meiotic cell cycles, it is absent in some mitotic cell divisions and therefore seemingly not indispensable in adult organisms [51,52]. Like cyclin D1, cyclin E is mainly present in proliferating cells. It is, however, also expressed in some non-proliferating cells like neurons [53]. As it is present in at least some GCs of all lesions studied, cyclin E seems to play a critical role in maintaining the cell cycle of the respective cells. This is also illustrated by the fact that a malfunction of cyclin E and related complexes can be found in most human tumours [54]. There are no published data available for the GCs of bony callus, sarcoidosis, sarcoid-like lesion, tuberculosis, foreign body granuloma, fibroid epulis, and brown tumour. The GCs of the aforementioned lesions showed between 30% and 100% immunoreactivity for cyclin E and may point to some remaining proliferative function. Live cell imaging has shown a continuous shuttling process of cyclin E from the nucleus (interacting with the replicative machinery of the cell) to the cytoplasm [55]. This may be a possible explanation for the exclusively cytoplasmatic staining of GCs in two samples of foreign body granuloma, while in one sample staining was strictly nuclear. Regarding cyclin E-expression of the two brown tumours analysed in one and the same patient, we noticed some differences. The brown tumour localised in the femoral region (sample 18a) showed staining in 30–60% of the GCs, while over 70% of GC in the brown tumour of the mandibula (sample 18b) stained positive. This may be due to differences in the cell cycle regulation of the two simultaneously occurring lesions. Brown tumours have long been regarded as a purely reactive lesion, the noxious agent being the systemically elevated parathyroid hormone. Recent findings reported by Turek et al. proved brown tumour to be one of the many pathologies with an activating KRAS-mutation, prompting the hypothesis that it is a true, mutation-driven neoplasm that requires a ‘second hit’ (i.e., hyperparathyroidism) to arise [18]. In many tumours, cyclin E is expressed higher than in the non-neoplastic presumed counterpart [56]. There have been both in vitro and in vivo studies that suggest that cyclin E plays a role as an oncogene causing genome instability and abnormality in cancer cells [57]. Although different neoplastic lesions have been discussed in the literature, there are no published data pertaining to GCs in aneurysmal bone cyst, chondroblastoma, non-ossifying fibroma, tenosynovial GC tumour or xanthogranuloma. As confirmed by this study, cyclin E has previously been detected in the GCs in GC tumour of the bone and is possibly involved in early replicative activity in the GCs [47].

p16 binds to and inhibits cyclin-dependent kinases (CDK) 4 and 6 that will otherwise bind to cyclin D1, leading to the phosphorylation of the retinoblastoma protein and enabling cell cycle progression. Thereby, the cell cycle is arrested at the G1/S-transition, which contributes to cellular senescence [58]. *CDKN2A*, the gene coding for p16, has been called the second most common tumour suppressor gene after *TP53*, the gene coding for p53, as it is altered or inactivated in a large variety of neoplasms [59]. Causing cell cycle arrest, p16 opposes the cyclins D1 and E and is expressed in low levels compared to the expression of these two molecules. This confirms the narrative that GCs can proliferate at the beginning, but are then arrested at the G1/S-transition of the cell cycle. In vitro studies have shown that p16 accumulates in cytoplasmatic organelles like vesicles and lysosomes as a reaction to cell starvation and autophagy [60]. This has led to the hypothesis that moderate levels of cytoplasmatic p16 are tolerated by the cell, without instantly impacting the cell cycle [60]. However, cell lines with no nuclear p16 expression have shown cytoplasmatic staining for p16, which is why exclusive cytoplasmatic staining is sometimes not considered a viable reaction [61]. Cytoplasmic localisation of p16 has been observed in our study in some GCs of GC tumour of the bone and aneurysmal bone cyst. For bony callus, sarcoidosis, sarcoid-like lesion, tuberculosis and brown tumour, there are no published comparative data regarding the expression of p16 by GCs. In our study, the GCs of these lesions were p16-negatvie or showed minimal immunoreactivity (up to 30%). This may point to a still partly active cell cycle in these GCs. The GCs of foreign body granuloma and fibroid epulis showed a medium to high immunoreactivity. As the GCs in these lesions were p21-immunoreactive, this may mean that the GCs of these lesions are involved in the process of senescence, i.e., that the cells are no longer proliferating. In the GC of GC tumour of the bone, p16 has been studied before and was found to show some nuclear and cytoplasmatic reaction, as is confirmed in our study [62]. This is linked to an apparent cell cycle arrest happening in GCs at the G1 stage [47]. Expression of p16 was lost in one sample of resected tissue of GC tumour of the bone, after having been detected in the incision biopsy (sample 25a and b). This can be explained by the distinct heterogeneity within the clinical and histological presentation of GC tumours of the bone. Different areas of the same tumour may have a different phenotype; there are even GC tumours of the bone without giant cells [63]. In some samples of aneurysmal bone cyst, p16 staining was strictly nuclear, while it appeared to be strictly cytoplasmatic in others. As discussed before, this can happen due to an accumulation of p16 in cytoplasmatic organelles and is sometimes not considered a viable reaction [61]. For the GCs of aneurysmal bone cyst, chondroblastoma and xanthogranuloma, there are no data available regarding p16-positivity. The GCs of tenosynovial GC tumour have been shown to express p16 before [64], which was confirmed by our study.

p21 is a CDK-inhibitor, binding to and inhibiting every cyclin/CDK-complex involved in the hyperphosphorylation of the retinoblastoma protein [29]. These events lead to cell-cycle arrest at the G1-stage [65]. Early studies revealed that the induction of *TP53* can occur through cellular stress like DNA-damage, and leads to increased transcription of *CDKN1A*, the gene coding for p21 [66]. Furthermore, p21 has been linked to osteoclastogenesis, since p21/p27 double knockout mice develop osteopetrosis [67]. Since the GCs of most osseous lesions studied here expressed some level of p21, this may trace back to the development of osteoclast-like GCs within them. In callus, p21 has previously been detected mostly in hypertrophic chondrocytes, and has been discussed as inducing apoptosis in these cells, enhancing osteoblast differentiation. [68] To our knowledge, the GCs in bony callus lesions have not yet been analysed with regard to p21 expression. The low but consistent levels detected in this study points to an enhanced bone turnover, with both active and pre-apoptotic osteoclast-like GCs. In granulomas of sarcoidosis, a focal increase of p21 was detected in cells of macrophage origin [69]. This may explain the long lifespan of granulomatous cells due to an inhibition of apoptosis. In our study, only a low percentage of GCs showed a reaction to p21. This may point to low levels of both proliferation and apoptosis in these cells. There are no data regarding p21-expression for GC of sarcoid-like lesion and tuberculosis. As suggested by their expression of p16, the GCs of foreign body granuloma and fibroid epulis seem to be in a state of senescence or reduced cell cycle activity, that is further proved by their expression of p21. However, there are yet no published data to compare these findings with. Brown tumour has been shown to be a neoplasia driven by *KRAS*-mutation [18]. The MAPK-pathway, for which *KRAS* is a defining molecule, constitutes a signalling cascade from the cell surface to the nucleus and is frequently mutated in various cancers [70]. Activation of the MAPK-pathway leads to induction of p21, thus causing cell cycle arrest [71]. This may explain our findings, showing a high expression of p21 in the GCs of one location of brown tumour (sample 18a). While occurring simultaneously in the analysed patient, the second analysed brown tumour (sample 18b) had a lower percentage of stained GCs, thus pointing to possible genetic differences of the two localisations in spite of the same underlying conditions. In the GCs of GC tumour of the bone, p21 has been shown to be increased compared to the mononuclear cell fraction [46]. As cyclin D1 and p21 often both stained positive in the GCs of various lesions, our study may indicate coregulated expression of these molecules. In samples 25a and 25b we noticed an inconsistency regarding p21 expression, as the incision biopsy of the lesion showed reactive GCs, while the resected tissue showed no reaction to p21. Since we excluded a technical failure due to intrinsic positive cells in the analysed tissue, the GCs were heterogenous in these tumours and the GCs within them. We have analysed the expression of p21 in aneurysmal bone cyst and shown that the mononuclear tumour component is p21-positive [62]. In the present study, the GCs of aneurysmal bone cyst showed some p21-immunoreactivity (30–70% of GC). However, there is an inconsistency with samples 32a and 32b, as the biopsies of the tumour were unreactive, while the later resected tissue was p21-positive in the GC compartment. This may be due to a heterogeneity of the expression of surface markers within the GCs of the same lesion and is in line with the heterogenous expression of p21 in the stromal compartment of aneurysmal bone cyst [62]. In chondroblastoma, p21 has been proved to be mainly expressed in GCs, as opposed to osteoblasts [20]. This was confirmed by our study and sustains the view of chondroblastoma being a lesion of the growth plate, with all molecules relevant for cell cycle present within the GCs. Non-ossifying fibroma, much like brown tumour, is constituted by an activating mutation of the MAPK-pathway [21]. Therefore, downstream activation of p21 as a result of a *KRAS*-mutation is a logical consequence. The GCs of tenosynovial GC tumour have been found to express p21 before [64]. This was attributed to enhanced p53-expression due to DNA-damage. The GCs of xanthogranuloma have not yet been analysed for p21-immunoreactivity. The lacking p21-expression is consistent with the equally absent p16-immunoreactivity in the GCs of this lesion. This means that the GCs have some level of cell cycle activity.

RANK is one of the three critical molecules for bone homeostasis studied here, along with RANK-L and OPG. RANK is a transmembrane protein that leads to osteoclast development in the presence of M-CSF (macrophage colony stimulating factor) [72,73]. The receptor is a member of the tumour necrosis factor receptor (TNFR) family and is present on osteoclast precursor cells, dendritic cells and activated T-cells [72,74]. A vesicular form of RANK is shed by maturing osteoclasts and bound by RANK-L in a reverse signalling process to stimulate osteoblast development and bone mineralisation [75]. RANK-knockout mice suffer from severe osteopetrosis because of a lack of osteoclasts, underlining the fundamental role of RANK for bone homeostasis [6]. RANK is activated by its ligand, receptor activator of nuclear factor κB-ligand (RANK-L), that is in turn secreted by osteoblasts and osteocytes, thus inducing osteoclastogenesis [76]. The GCs of bony callus and brown tumour are considered to be osteoclast-like GCs that express similar surface markers to osteoclasts, including RANK [3]. For the GCs of sarcoidosis and tuberculosis, there are no data regarding their RANK-immunoreactivity. In vitro, Langhans-resembling GCs have been generated through the treatment of macrophages with RANK-L, along with lipopolysaccharide and interferon-γ stimulation, that are known to lead to a differentiation of macrophages into Langhans-GCs of tuberculosis [77]. These cells resorb bone and were responsive to RANK-L-treatment, confirming our data. In the GCs of foreign body-granuloma, RANK-positivity has been shown before [78]. The GCs of sarcoid like lesion, and fibroid epulis have not been studied with regard to their RANK-positivity before. Considering the ties between immunology and RANK, the expression pattern of this antigen is an intriguing expansion of the existing data on these GC of reactive lesions. The osteoclast-like GCs in GC tumour of the bone have been shown to express RANK, as their stimulation by RANK-L and the subsequent high rate of bone resorption is a key feature of pathogenesis in this neoplasia [79]. The GCs of aneurysmal bone cyst have been found to express RANK [80], as confirmed by our findings. RANK-expression is also a feature of the GCs in chondroblastoma [81], as has been confirmed by our study. As non-ossifying fibromas include osteoclast-like GCs, [19] they also show immunoreactivity for RANK. The GCs of tenosynovial GC tumour have been shown to express osteoclast-like surface markers [82]. A high level of RANK has been found in the GC-rich specimens of tenosynovial GC tumour [83]. Touton GCs featured in xanthogranuloma have not yet been analysed with regard to their RANK-expression. The results of our study, albeit with a very small sample size, suggest a possible inherent difference to the other, bone-associated GCs of neoplastic lesions.

RANK-L is the ligand for RANK and constitutes the only ligand that binds to this receptor. It also binds to the decoy-receptor OPG with high affinity [6]. RANK-L is bound to the membranes of osteoclastogenesis-inducing cells, including osteocytes, osteoblasts and lymphocytes [84]. It is also present on activated T-cells; suggesting that these cells can trigger osteoclastogenesis [85]. In vivo, it has been shown that osteocytes, and not osteoblasts, are the predominant source of RANK-L [84], with osteocytes ceasing to express the protein as they become embedded in the bone matrix [86]. Huang et al. suggest that RANK-L is the sole most important factor of osteoclastic development, as recombinant RANK-L can induce osteoclastogenesis in the absence of osteoblasts/osteocytes [87]. Consequently, RANK-L-deficient mice develop severe osteopetrosis as a result of a lack of bone resorption by osteoclasts [88]. The RANK/RANK-L-axis was shown to also affect the development of bone metastases and mammary cancer, broadening the scope of relevance for these molecules [76]. In general, RANK-L is present in cell types that induce the differentiation of multinucleated GC and binds to RANK that is expressed on the surface of these cells (as in the osteoblasts and osteoclasts of physiological bone). RANK-L is not found to be expressed by immunohistochemistry on GCs, as has been reproduced by our study. However, in some lesions, we found RANK-L on the surface of a small percentage of GCs, where it is most likely bound by RANK. This finding has been shown in GC tumours of the bone before [79]. Most osteoclast-like GCs in GC tumour of the bone express RANK-L [86]. In bony callus, a relatively high level of RANK-L was determined at the end of primary bone formation [89]. However, this increased detection of RANK-L is not specific to GCs and is likely due to an elevated bone metabolism at that stage, thus reflecting a dependence on functional activity. Additionally, activated T-cells that express RANK-L are part of the initial inflammatory response following a fracture, and may contribute to the rise of RANK-L levels in these tissues [85,89]. It is well documented that osteoblasts in spinal tuberculosis show increased levels of RANK-L, which entails a heightened bone resorption by osteoclasts [90]. However, to our knowledge osteoclasts in bone tuberculosis and Langhans GCs have not been subjected to these analyses so far. For the GCs of sarcoidosis, sarcoid-like lesion, and brown tumour, there are no data regarding RANK-L expression. RANK-L was detected as being expressed by the spindle-like tumour cells of GC tumour of the bone before, while the GCs were shown to be RANK-L negative [79]. Among other secreted cytokines like macrophage colony stimulating factor (MCSF) [3], elevated expression of RANK-L leads to a high rate of bone resorption due to the stimulation of osteoclast-like GC [87]. This connection has been exploited therapeutically with the introduction of the specific RANK-L-antibody Denosumab that prevents RANK-L-RANK-interaction in GC tumour of the bone and leads to a significant decline in GCs and bone resorption [91]. Mononuclear cells of aneurysmal bone cysts express RANK-L in a similar quantity to GC tumour of the bone and are thought to entail the same osteolytic effects in the lesion, which was utilised for a case study using Denosumab in the treatment of aneurysmal bone cyst [80]. In previous studies, RANK-L was absent from GCs [80], while it was shown in a low percentage of GCs in the data presented here. Likewise, the GCs of chondroblastoma have been shown to lack expression of RANK-L, while mononuclear cells in the tumour are RANK-L positive [92]. In tenosynovial GC tumour, RANK-L expression was detected in the stromal cells, but not in multinucleated GC [83]. For the GCs of non-ossifying fibroma and xanthogranuloma, there are no data regarding the expression of RANK-L. All in all, the expression of RANK-L primarily in the mononuclear cell population of GC-containing neoplastic lesions supports the thesis that osteoclast-like GCs are recruited in a similar way as osteoclasts in physiological bone, that is, by stimulation of RANK-L [14].

OPG acts as a soluble decoy receptor for RANK-L and prevents interaction with osteoclast precursors, thereby inhibiting the formation and activation of osteoclasts [72]. Like RANK-L, OPG is expressed by osteoblasts and osteocytes, but lacks the transmembrane domain of RANK [6]. OPG-depleted mice suffer from low trabecular bone density and resulting fractures, illustrating the importance of OPG in bone development [93]. Downregulation of OPG is essential for the development of osteoclasts, even when RANK-L is present, making the RANK-L/OPG-ratio a determining factor for osteoclastogenesis [94]. In bony callus, OPG is known to be present at different phases of healing, particularly during cartilage formation [89]. This finding is however not specific to GCs. There are no data concerning the OPG-expression for GC of sarcoidosis, sarcoid-like lesion, fibroid epulis and brown tumour. Levels of circulating OPG were shown to be decreased in patients with spinal tuberculosis compared to non-spinal tuberculosis, which is viewed as a reason for disrupted bone homeostasis in these patients [95]. However, there are no data available concerning the OPG-expression of Langhans-cells. In foreign body granuloma, OPG was not found in the majority of GCs [78]. In our study, 30–70% of foreign body GCs were OPG-positive, which constitutes a decrease compared to most other lesions studied. Both in GC tumour of the bone [87] and in aneurysmal bone cyst [96], OPG-expression has been shown in the GC compartment before and is now confirmed by this study. In chondroblastoma, OPG has been detected in the stromal cells of the tumour [92], a finding that has been extended to the GC fraction in this study. In tenosynovial GC tumour, OPG-expression has been detected both in the general tissue [83], and in GCs specifically [22]. For the GCs of non-ossifying fibroma and xanthogranuloma, there are no data regarding the OPG-expression.

Osteonectin is a glycoprotein present in the extracellular matrix of bone and a variety of non-mineralised tissues [97]. During cell differentiation and early bone formation in immature bone, osteonectin is expressed by osteoblasts, whereas expression decreases in mature osteoblasts and adult bone [97,98]. The glycoprotein is expressed by macrophages [99], but has not been reported to be expressed by osteoclasts [100]. Osteonectin is thought to be imperative for the assembly of extracellular matrix and basement membranes [101]. Osteonectin-knockout mice lack fibrillar collagen I, therefore osteonectin mediates the processing and integration of collagen into the extracellular matrix [102,103]. Further, in osteonectin-knockout mice, there is a reduced number of osteoblasts and osteoclasts, resulting in a lower rate of trabecular bone formation and in early-onset osteopenia [104]. As osteoclastogenesis is mainly stimulated by osteoblasts, the reduced number of osteoblasts with a shorter lifespan may result in the development of fewer osteoclasts [98]. In patients with osteogenesis imperfecta, osteoblasts exhibit decreased levels of osteonectin [105], and a mutation in the osteonectin gene was found to lead to a severe form of osteogenesis imperfecta [106]. Osteonectin has been described as accelerating some cancers while inhibiting others and seems to be a requirement for metastasis in mammary carcinomas [99]. Elevated levels of osteonectin have been documented in bony callus, albeit not in the GC compartment [107]. This is in line with the greater level of osteonectin present in the osteoblasts of developing bone, as compared to mature bone [98]. For the GCs of sarcoidosis, sarcoid-like lesion, tuberculosis and fibroid epulis, there are no data concerning the osteonectin-immunoreactivity. In osteonectin-null mice, a thinner capsule and fewer GCs associated with a foreign body reaction could be observed [108]. The GCs themselves were not reported to be osteonectin-positive. The osteoclast-like GC of brown tumour were shown to be osteonectin-negative [109]. In GC tumour of the bone, GCs were found to be osteonectin-negative, while the spindle-cell component of the tumour was shown to be osteonectin-positive [110]. In aneurysmal bone cyst, the osteonectin gene *SPARC* has been identified as one of the possible fusion partners in the characteristic *UPS6*-rearrangement of the lesion [111]. However, GCs have not been discussed with regard to osteonectin-positivity yet. As an intrinsic positive control has always been ensured, the differences within aneurysmal bone cyst samples in our results may be due to tumour-inherent heterogeneity. In chondroblastoma, mononuclear cells were shown to express osteonectin, whereas GCs were osteonectin-negative [109]. The osteonectin-positivity of up to 70% of the GCs in our study is therefore a novel finding. There are no data concerning the expression of osteonectin for GC of non-ossifying fibroma, tenosynovial GC tumour and xanthogranuloma.

Like osteonectin, osteopontin is considered to be a matricellular glycoprotein that interacts with different cell types, proteins, molecules, and soluble factors of the extracellular matrix [112]. It comprises about 2% of the total non-collagenous protein bone mass [113]. Secreted by mature osteoblasts, osteopontin serves as a marker for osteoblastic differentiation [114]. It is also present in osteoclasts, and to varying degrees in osteocytes and chondrocytes [113]. Osteopontin is likely required for osteoclastogenesis [115]. In endothelial cells, osteopontin activates the NF-κB-pathway and therefore modulates inflammation [116]. The molecule can influence macrophage differentiation, infiltration and cytokine release, thereby influencing the inflammatory process [113]. It is also crucial for cellular immunity, as osteopontin-null mice have severely reduced immunity to bacterial and viral infections [117]. Osteopontin is essential for bone remodelling, but not for bone development [113]. In states of oestrogen-deficiency, osteopontin-knockout mice suffer less bone resorption than wildtype mice, linking osteopontin to the development of osteoporosis [118]. The molecule is overexpressed in many tumours, as it mediates the migration and adhesion of many cell types and has been particularly well studied in connection with tumour cell invasion and calcification in breast cancer [113]. Conversely, osteopontin has also been shown to slow tumour growth and limit tumour cell line survival [119]. Osteopontin has been shown to be expressed in the osteoclast-like GCs of callus, as well as in osteocytes and osteoprogenitor cells [120]. In osteopontin-knockout mice, fracture healing was shown to be significantly impaired, with decreased osteoclast function and reduced callus stability [121]. In sarcoidosis, the plasma levels of osteopontin are elevated [122], and osteopontin is expressed by T-lymphocytes in sarcoidosis granulomas [123]. As a manifestation of their severely decreased cellular immune response, osteopontin-knockout mice do not develop sarcoid granulomas [117]. To our knowledge, there are no published data concerning the osteopontin expression of GCs in sarcoidosis and sarcoid like lesion. In bovine tuberculosis granuloma, osteopontin has been shown to be expressed strongly by Langhans GCs [124], while in one study on human tuberculosis, Langhans GCs did not show osteopontin-positivity [125]. The GCs of foreign body granuloma were shown to strongly express osteopontin [126]. The GCs of different GC-containing lesions of the mouth similar to fibroid epulis were shown to be osteopontin-positive [127]. For brown tumour, there are no data regarding the osteopontin-expression of osteoclast-like GCs. In GC tumour of the bone, stromal cells were reported to be osteopontin-positive, while GC were osteopontin-negative [110]. This is at variance with the data of our study, where the GCs in GC tumour of the bone were osteopontin-positive throughout. This may be explained by their osteoclast-like nature and the fact that osteoclasts express osteopontin [113]. For the GCs of aneurysmal bone cyst, chondroblastoma (cytoplasmatic), and non-ossifying fibroma, there are no data referencing osteopontin-expression in the GCs of these lesions. The lacking consistency in the results of samples 32a and 32b (biopsy and resected tissue of an aneurysmal bone cyst) may again be explained by the heterogenic distribution of cells expressing certain markers in this type of lesion. Regarding the cytoplasmatic localisation of osteopontin-staining in one chondroblastoma, an intracellular version has been described early on that is often associated with the CD44 receptor [114]. The GCs of tenosynovial GC tumour were previously shown to express osteopontin [22]. The presence of osteopontin in xanthogranuloma tumour tissue was shown before, however the GCs were not specifically analysed [128]. Since osteopontin-positive macrophages are known to accumulate lipids and form the so-called Touton GC [3,129], it is possible that osteopontin-expression may be lost during Touton cell formation.

TRAP is an iron-containing enzyme present in macrophages, dendritic cells and epithelial cells in many different tissues [130,131]. Highly expressed in osteoclasts, it has been used as a marker for these cells for decades [132]. TRAP is secreted by osteoclasts, and secretion and bone resorption by osteoclasts are positively correlated [133]. TRAP has also been proposed to regulate osteoclast activity through the dephosphorylation of osteopontin, that then no longer mediates cell attachment to the surface of the bone [134]. The immunoregulatory function of osteopontin may also be influenced by TRAP [135]. In the endochondral ossification of developing bones, TRAP plays a significant role in mineralisation, and TRAP-knockout mice suffer from osteopetrosis due to a reduction in osteoclast function [136]. The phenotype of the skeleton, particularly that of the long bones, is also significantly altered in TRAP-knockout mice [135]. Through activation of osteoblast differentiation, TRAP was discussed as additionally playing a role in bone formation [137]. However, there are also data that suggest downregulation of osteoblast activity by TRAP [135]. TRAP levels are increased in conditions like hyperparathyroidism and osteoporosis [135]. In patients with breast cancer and progressive bone metastases, TRAP serum levels are significantly increased, making TRAP a possible marker for the progression of bone metastases [138]. Osteoclast precursor cells and osteoclasts in bony callus have been shown to express TRAP [139]. In the GCs of sarcoidosis, TRAP was strongly and specifically expressed [140]. It was detectable in the inflammatory macrophages as well, strengthening the case for a macrophage biomarker for different kinds of chronic inflammations. In a study comparing the TRAP-expression of the GCs in tuberculosis and sarcoidosis, the GCs of tuberculosis stained TRAP-positive to a significantly stronger degree [141], as has been confirmed by this study. For the GCs of sarcoid-like lesion and fibroid epulis, there are no data concerning TRAP-expression. The GCs of foreign body granuloma were shown to express TRAP to a significant degree [142]. It has been demonstrated that the GCs of brown tumour were TRAP-positive when localised near an area of bone matrix, while inset GCs did not express TRAP [143]. Although our data do not corroborate these spatial differences, the findings may reflect the fact that only a certain percentage of GCs expressed TRAP in our study. In the GCs of GC tumour of the bone, TRAP-staining has been confirmed and emphasises the relation of osteoclast-like GCs and osteoclasts [86,110]. GC of aneurysmal bone cyst also express TRAP, underlining their similarity to osteoclasts [96]. For the GCs of chondroblastoma, similar assumptions have been made, as they are also TRAP-positive [144]. The GCs of tenosynovial GC tumour have also been shown to be TRAP-positive [64]. For the GCs of non-ossifying fibroma and xanthogranuloma, there are no data regarding TRAP-expression.

RUNX2 is a member of the RUNX family of DNA-binding transcription factors and is expressed by osteoblasts and chondrocytes [145]. Studies performed on RUNX2-knockout mice confirm a critical role of the transcription factor for the differentiation of these cell types [146]. As a key element of osteogenesis among other transcription factors, RUNX2 directs mesenchymal stem cells toward a differentiation into osteoblasts and inhibits their development into mature osteoblasts and osteocytes [147]. Studies in transgenic mice have shown that RUNX2-levels need to be downregulated in order for immature osteoblasts to mature [36]. At the other end of bone remodelling, RUNX2 has also been shown to facilitate bone resorption, as it was found to be expressed in macrophages and osteoclasts and promote osteoclastogenesis [148]. RUNX2-knockout mice completely lack osteoblasts [149], endochondral ossification and the expression of osteopontin [146]. Besides its role in chondrocyte differentiation, RUNX2 also seems to play a role in the development of osteoarthritis, as RUNX2-knockout mice are resistant to cartilage degradation [150]. RUNX2 is associated with many cancers and has been shown to be a risk factor for invasion and metastasis in different carcinomas [151]. Specifically, it has been described in breast cancer and prostate cancer with bone metastasis [36]. Overall protein expression of RUNX2 in bony callus has been shown to be extensive, even though GCs have not specifically been addressed [152]. RUNX2 has also been shown to have a dramatic positive impact on bone wound healing [153]. There are no data regarding the RUNX2-expression of GCs in sarcoidosis, sarcoid like lesion, foreign body granuloma, fibroid epulis or brown tumour. In patients with tuberculosis and accompanying ossification of the lung, RUNX2 has been shown to be present in macrophages, leading to the osteogenic differentiation of mesenchymal stem cells [154]. However, the GCs of tuberculosis have not specifically been discussed regarding their RUNX2 expression pattern. RUNX2 has been shown to be highly expressed in GC tumours of the bone [155]. However, RUNX2-expression reported in the literature is mainly localised in the mononuclear stromal cells; in this localisation, the transcription factor has been proposed to play a role in the osteolytic activity within GC tumours of the bone [156]. The heterogenous expression of RUNX2 within GC tumours of the bone in our study may be due to an unequal distribution of mature osteoclast-like GC, and areas of osteolysis driven by RUNX2 [157]. The same reason may apply to the heterogeneity of RUNX2-expression in the GCs of aneurysmal bone cyst. Remarkably, the *RUNX2* gene has been discovered by next generation sequencing to be one of the fusion partners of the gene encoding ubiquitin-specific peptidase 6 (USP6), possibly contributing to the osteoblastic as well as osteolytic features of this neoplasia. RUNX2-expression has been shown in the neoplastic, mononuclear compartment of chondroblastoma, but not in the GCs, confirming our data [158]. There are no data regarding RUNX2-expression for GC of non-ossifying fibroma, tenosynovial GC tumour or xanthogranuloma.

CD68 is a transmembrane protein that is heavily glycosylated and belongs to the family of lysosomal-associated glycoproteins (lamp-1). Unlike the other broadly expressed members of the lamp-family, CD68 is mainly present on the lysosomal and plasma membranes of macrophages and other cells of the monocytic lineage, like osteoclasts [37]. However, CD68 has been shown to occur in a variety of other haematopoietic and non-haematopoietic cell types, leading to the hypothesis that CD68 is not a specific marker for monocyte-derived cells, but is merely present in higher concentrations in these cell types [159]. Although its function is still largely unknown, CD68 has been linked to the regulation of T-cell-activation [160]. Furthermore, CD68 seems to play a part in osteoclast development, since CD68 knockout-mice have a higher bone volume with poor mineralisation and less efficiently bone-resorbing osteoclasts [161]. Expression of CD68 has been linked to different types of macrophages (M1 and M2) in bony callus and is used as a marker for monocyte derived cells [162]. Our data confirm CD68-expression in GCs [14]. In sarcoidosis, GC shave been shown to be CD68-positive, pointing to their derivation from inflammatory macrophages [140]; our data confirm this. In sarcoid-like lesion in renal cancer, epithelioid cells of the granuloma stained CD68-positive [16]. In tuberculosis, CD68 has been used as a marker for phagocytotic cells before [163]. Both the Langhans-type GCs of sarcoidosis and tuberculosis and the foreign body GCs are CD68-positive throughout [12]. CD68-reactivity has additionally been shown in GC cholesterol granulomas, a subtype of foreign body granulomas [164]. In the case of fibroid epulis, there are no data regarding the immunoreactivity of GCs for CD68. In our study, above 70% of GCs showed positive staining, thereby confirming the monocyte lineage of these GCs. The GCs in brown tumour of both localisations studied here showed above 70% immunoreactivity for CD68, confirming the monocytic lineage of these osteoclast-like-GCs. GC in GC tumour of the bone have frequently been found to show immunoreactivity for CD68 [165,166,167]; this was confirmed by our study, as all analysed GCs of these lesions stained positive for the antigen. Since the number of GCs in GC tumour of the bone is determined by other factors like RANK-L, CD68-expression confirms their differentiation as osteoclast-like GCs of a monocyte origin. Similarly, the GCs of aneurysmal bone cyst are known to express CD68 and therefore present an osteoclastic phenotype [96]. The same is true for GCs of chondroblastoma, that also showed positive immunoreactivity for CD68, both in the literature [168] and in our study. For the GCs in non-ossifying fibroma, there are no data with regard to CD68-immunoreactivity. However, as in most other lesions, GCs showed positive staining in over 70%, implying the same lineage for all of them. As confirmed by our data, the GCs in tenosynovial GC tumour have also been documented to shown CD68-immunoreactivity [64,169], as do macrophages in the tumour tissue [170]. In xanthogranuloma, CD68 is used to confirm the presence of so-called Touton giant cells (a particular type of GC that is seen in lesions of a high lipid content) and therefore helps with the diagnosis of this disease [171].

CD163 is a scavenger receptor for haemoglobin (Hb) molecules and the haemoglobin-haptoglobin-complex (Hb-Hp), that leads to their phagocytising and degradation upon haemolysis or haemorrhage, reversing the pro-inflammatory effect of these processes [172]. CD163 is present on the surface of CD14-positive monocytes and tissue macrophages [173]. Notably, CD163 in other monocyte-derived cells like dendritic cells is absent [38]. Although macrophages do fuse to form GCs, the surface antigen CD163 is not a feature of these GCs and has been found to be absent from osteoclasts, osteoclast-like GCs, and macrophage polykaryons like Langhans GCs and foreign body GCs [14]. One explanation is that CD163 is present only in a sub-population of macrophages and macrophage-derived cells but is lost in the fusion process of multinucleated GCs [38].

Langerin (CD207) is a transmembrane, C-type lectin receptor that binds the carbohydrate fraction of many pathogens [39]. These langerin-positive cells then migrate to lymph nodes, where langerin is involved in antigen processing and presentation to T cells, thus contributing to cellular immunity [174]. A role of langerin in the formation of so-called Birbeck granules has been suggested; these granules are thought to be residuals of membranous structures and are localised within the cytoplasm of so-called Langerhans cells that are thought be involved in antigen presentation [175,176]. Although dendritic cells are part of the monocyte-macrophage system, they belong to a distinct subcategory with different surface markers from macrophages or multinucleated GCs. Therefore, our findings of consistently langerin-negative GCs are in line with data from the literature and supplement the literature.

## 5. Conclusions

**Figure 2:**
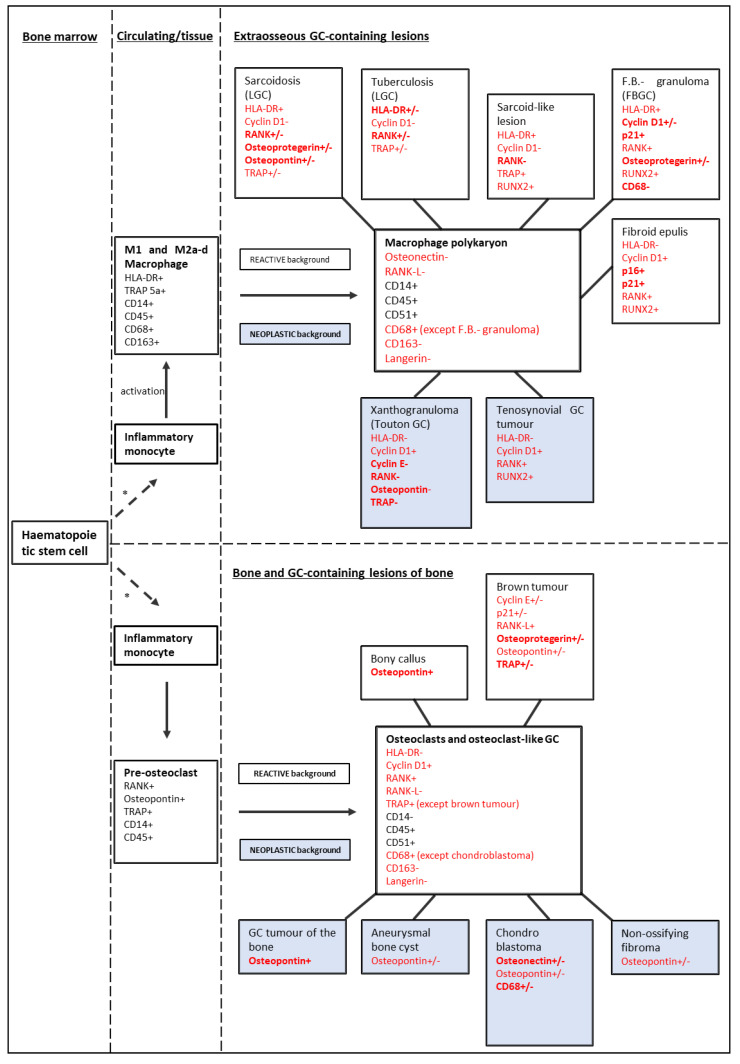
Expression profile of various GC and their precursors. Black antigens: data from literature (Brooks [3], Maggiani [14], Atkins et al. [177], Takeshita et al. [178]); red antigens: data confirmed or established by this study. In bold: discriminating antigens expressed by up to two lesions. In standard font: non-discriminating antigens. Above 70% immunoreactive GC: +; 30−70% immunoreactive GC: +/−; up to 30% immunoreactive GC: −. Blue background lesions: neoplastic lesions; white background lesions: reactive lesions. * = With various differentiating steps (Gordon et al. [11]). M1: inflammatory macrophage; M2a-d: inflammation resolving macrophage; LGC: Langhans giant cell; F.B.- granuloma: foreign-body granuloma; FBGC: foreign body giant cell.

While analysing the expression pattern of GCs of different antigen categories in the multitude of lesions studied here, a broad profile emerged (see Figure 2). Most notably, the dividing line of GC phenotypes does not pass in between the GCs of reactive and neoplastic lesions but rather between the osteoclast-like GCs of GC-rich lesions of bone and macrophage polykaryons of extraosseous GC-containing lesions. This concept has been described before [14]; and was confirmed by our analysis. A common macrophage origin of many GCs was demonstrated by the CD68-positivity of most GCs and the subsequent derivation from the macrophage path was illustrated by the CD163-negativity of all analysed GCs. Osteoclast-like GCs were HLA-DR-negative throughout, while some macrophage polykaryons were HLA-DR-positive. The distribution of cell cycle-associated antigens within most lesions suggests a mechanism of GC formation that possibly surpasses a mere fusion process. The expression of cyclin D1 and cyclin E (cell cycle promoting antigens) and the lacking or lower expression of p16 and p21 (cell cycle inhibiting antigens) raise the question whether mitosis is still a part of GCs. Acytokinetic cell division may be partly involved in GC formation, as has been reported in the GCs of myxofibrosarcoma [7].

Notably, the GCs of xanthogranuloma were negative for molecules like cyclin E, RANK, and osteopontin, that are common among the GCs of the other analysed lesions. TRAP was confirmed as a general marker of all GCs, except xanthogranuloma. This unique profile may be explained by the specific formation mechanism of the Touton-GCs of xanthogranuloma: they are derived from activated blood monocytes that have phagocytised large amounts of lipids, to the point of inducing hypocholesterinaemia in patients [129]. On top of the lesions standing out from the crowd, histologically similar lesions like chondroblastoma and tenosynovial giant cell tumour, that might be mixed up on histological grounds only [169] now are defined through some discriminative immunohistological differences in the GC compartment, that may therefore facilitate diagnosis of these lesions. Most notably, the GCs of tenosynovial giant cell tumour are RUNX2-positive, while the GCs of chondroblastoma are RUNX2-negative throughout. For some pathologies including sarcoid-like lesion, few data concerning GCs have been presented before. As sarcoid-like lesion is often associated with malignant pathologies, it is crucial to distinguish this lesion from benign sarcoidosis in order to treat the underlying neoplasm as soon as possible [179]. Our data suggest a few subtle differences in the antigen expression pattern of sarcoidosis and sarcoid-like lesion, that may be of help in differentiating the two lesions in the future. The difference in the RANK-expression of GCs is most apparent, as the GCs of sarcoidosis are up to 70% RANK-positive, while the GCs of sarcoid-like lesion show no reaction at all. Our results confirm the heterogenous nature of some GC-rich lesions [142], with slightly different phenotypes being detected in some biopsies and respective resected tissues.

As osteoclast-like GCs show largely similar expression profiles to osteoclasts, the specific anti RANK-L-antibody Denosumab may be beneficial in pathologies beyond GC tumour of the bone, such as aneurysmal bone cyst and chondroblastoma, for which off-label uses have been reported [14]. Recurring cases of non-ossifying fibroma and tenosynovial GC tumour may profit from Denosumab.

A weakness of our study is its purely descriptive nature, albeit at a detailed level. In addition, the sample size for many of the rarer analysed lesions is small and therefore only suitable for portraying a tendency. For these reasons, the data present the platform for further studies, and an even broader panel of analysed molecules:

In physiological bone, osteoclast formation depends on the presence of macrophage-colony stimulating factor (M-CSF) [3]. However, in giant cell tumour of the bone, it has been shown that there are other growth factors like vascular endothelial growth factor (VEGF) and hepatocyte growth factor (HGF), that stimulate osteoclastogenesis, even in the absence of M-CSF [180]. Following a combined stimulation of monocytes with M-CSF and other growth factors, the resulting GC are more aggressive and resemble those of locally aggressive lesions like giant cell tumour of the bone, thus linking angiogenesis and osteolytic destruction in these lesions [180,181].

Consequently, it is of interest to study the prevalence of and susceptibility to VEGF and other growth factors in the GCs of different lesions, as giant cell tumours of the bone have been shown to be sensitive to antiangiogenetic tyrosine kinase inhibitor treatment combined with Denosumab [182].

To sum up, GCs derive from various lines of differentiation and seem to reflect the respective microenvironment (i.e., cytokines, matrix components), origin, and function. The antigen expression pattern of GCs mirrors these differences, gives further insight into the common and distinctive features of this cell type, and provides a platform for further studies.

## Figures and Tables

**Table 1:** Description of the analysed lesions, the type of giant cell (GC) they contain, and the GC’s function.

Lesion	Description	Type of GC	Function
Bony callus [15]	Primary bone formation in fracture healing	Osteoclast	Bone remodelling
Sarcoidosis [4]	Systemic granulomatous disorder with noncaseous epithelioid cell granulomas	Langhans GC	Unknown
Sarcoid-like lesion [16]	Malignancy-associated granulomatous reaction commonly found at cancer site or in regional lymph nodes	Macrophage polykaryon	Unknown
Tuberculosis [4]	Infectious disease caused by *Mycobacterium tuberculosis* with caseous granulomas	Langhans GC	Pathogen phagocytosis
Foreign body granuloma [9]	Local reaction to a foreign object or substance within the tissue that cannot be phagocytised	Foreign body GC	Resorption of foreign material
Fibroid epulis [17]	Fibrous hyperplasia of the gingiva	Macrophage polykaryon	Unknown
Brown tumour [18]	Bone tumour that develops in response to long-lasting hyperparathyroidism coupled with *KRAS*-mutation and activated MAPK-pathway signalling	Osteoclast-like GC	Bone resorption
Giant cell tumour of the bone [19]	Locally aggressive bone tumour driven by *H3F3A*-mutatoin	Osteoclast-like GC	Bone resorption
Aneurysmal bone cyst [19]	Benign bone tumour driven by *USP6*-rearrangement	Osteoclast-like GC	Bone resorption
Chondroblastoma [20]	Benign bone tumour with cartilage features, driven by *H3F3B*-mutation	Osteoclast-like GC	Bone and cartilage resorption
Non-ossifying fibroma [21]	Benign bone tumour driven by *KRAS*-mutation and activated MAPK-pathway signalling	Osteoclast-like GC	Bone resorption
Tenosynovial giant cell tumour [22]	Group of lesions derived from tendon sheaths, synovia or bursae	Macrophage polykaryon	Unknown
Xanthogranuloma [23]	Benign cutaneous non-Langerhans cell histiocytosis	Touton GC	Lipid phagocytosis

**Table 2:** Clinical data of all analysed samples.

Sample No.		Age	Sex	Localisation	Dimensions in cm
	Reactive lesions				
	Bony callus (*n* = 4)				
1		15	Male	Left elbow joint	2.5 × 1.5 × 0.4
2		44	Male	left tibia	1.2 × 1.2 × 0.5
3		52	Male	Proximal radius	3 × 2.5 × 0.5
4		14	Female	Right deltoid muscle (ossificated)	6 × 6 × 2.5
	Sarcoidosis (*n* = 3)				
5		66	Female	Liver	Core biopsy (0.3 × 1.4)
6		28	Male	Epididymis	2.2 × 1.6 × 1.4
7		36	Male	Lymph node	2.2 × 1.5 × 0.5
	Sarcoid like lesion in rectal cancer (*n* = 1)				
8		62	Male	Paracardial lymph node	1.5 × 1.2 × 0.5
	Sarcoid like lesion in pancreatic corpus carcinoma (*n* = 1)				
9		63	Female	Lymph node common hepatic artery	2.5 × 2.5 × 1
	Tuberculosis (*n* = 3)				
10		88	Male	Thoracic vertebra 5	1.3 × 0.3 × 0.3
11		86	Female	Soft tissue distal forearm	3.5 × 3 × 1
12		80	Female	Trabecular bone	2.7 × 2.8 × 1.9
	Foreign body granuloma (*n* = 3)				
13		31	Female	Left parasternal cutaneous and subcutaneous tissue	4.6 × 1 × 1.1
14		94	Female	Adnexa of uterus	3 × 3 × 2
15		79	Female	Scar tissue of right mastectomy	Three core biopsies 1 × 1.6
	Fibroid epulis (*n* = 2)				
16		25	Female	Left auricle	2.5 × 1.7 × 0.7
17		61	Male	Gingiva	1.1 × 0.5 × 0.5
	Brown tumour (*n* = 2)				
18a		20	Male	Tumour right femur	2.2 × 1.9 × 0.6
18b		20	Male	Tumour right mandible	1.6 × 1.2 × 0.4
	Neoplastic lesions				
	Giant cell tumour of the bone (ICD-O 9250/1) (*n* = 11)				
19		17	Male	Left head of the fibula	7 × 7 × 3
20		38	Male	Left distal femur	3 × 2.5 × 1
21		38	Male	Left distal femur	5 × 5.2 × 1.4
22		30	Female	Lateral condyle of right femur	2.2 × 2 × 0.4
23		31	Male	Right proximal fibula	2 × 2 × 0.5
24a		20	Male	Core biopsy right proximal tibia	Four core biopsies, 2 cm
24b		20	Male	Resected tissue right proximal tibia	4 × 4 × 2.5
25a		18	Female	Incision biopsy right head of the tibia	1.4 × 1.6 × 0.4
25b		18	Female	Resected tissue right head of the tibia	7 × 4 × 2
26a		42	Male	Right head of the fibula	1.9 × 0.8 × 0.3
26b		46	Male	Local recurrence in right head of the fibula	2.5 × 2.5 × 0.5
	Aneurysmal bone cyst (ICD-O 9260/0) (*n* = 7)				
27		9	Male	Rib	2.2 × 1.4 × 1.4
28		19	Male	Fibula	0.5 × 0.3 × 0.2
29		25	Female	Left proximal tibia	4.5 × 3.4 × 0.8
30		27	Male	Lower thoracic spine	3.5 × 2.7 × 1.4
31		22	Male	Metacarpal bone of left hand	1.3 × 0.6 × 0.5
32a		45	Female	Incision biopsy left thumb	0.9 × 0.6 × 0.2
32b		45	Female	Left thumb	1.5 × 1.7 × 0.3
	Chondroblastoma (ICD-O 9230/0) (*n* = 3)				
33		15	Male	Medial condyle of left femur	1.5 × 0.9 × 0.8
34		13	Female	Right tibia	1 × 0.7 × 0.4
35		21	Male	Right fifth rib	1 × 0.7 × 0.5
	Non-ossifying fibroma (8830/0) (*n* = 3)				
36		10	Female	Right distal femur	3.5 × 2.5 × 1.7
37		12	Male	Cancellous bone	2.5 × 2 × 0.5
38		16	Male	Left distal tibia	4.5 × 4.4 × 0.6
	Tenosynovial giant cell tumour (ICD-O 9252/0) (*n* = 3)				
39		45	Male	Toe	2.9 × 1.5 × 1.1
40		53	Male	Left knee	3.5 × 1.5 × 0.4
41		20	Female	Cervical vertebra	1.5 × 0.5 × 0.5
	Xanthogranuloma (*n* = 1)				
42		52	Male	Eyelid cyst	0.5 × 0.3 × 0.2

**Table 3:** Antibodies used for immunohistochemistry in this study.

Antibody	Clone	Cat#	Dilution	Pretreatment	Manufacturer	Location
Anti-human leukocyte antigen (HLA)-DR (mouse)	1B5	-	1:1000	Microwave	Moldenhauer Heidelberg	Heidelberg, Germany
Anti-Cyclin D1 (rabbit)	EP12	M3642	1:25	Steamer pH9	Dako/Agilent Technologies, Inc.	Santa Clara, California, USA
Anti-Cyclin E (mouse)	HE12	sc-247	1:75	Steamer pH 8	Santa Cruz Biotechnology, Inc.	Dallas, Texas, USA
Anti-p16 (mouse)	1D7D2	MA5-17054	1:400	Microwave	Thermo Fisher Scientific	Waltham, Massachusetts, USA
Anti-p21 (WAF1/Cip1) (mouse)	SX118	M7202	1:25	Steamer pH8	Dako/Agilent Technologies, Inc.	Santa Clara, California, USA
Anti-receptor activator of nuclear factor κB (RANK) (rabbit poly)	TNFRSF 11A	NBP1-85771	1:100	Steamer pH 9	Novus Biologicals, LLC	Centennial, Colorado, USA
Anti-receptor activator of nuclear factor κB ligand (RANK-L) (mouse)	12A668	NB100-56512	1:300	Steamer pH8	Novus Biologicals, LLC	Centennial, Colorado, USA
Anti-Osteoprotegerin (OPG) (mouse)	98A1071	NB100-56505	1:400	Steamer pH 8	Novus Biologicals, LLC	Centennial, Colorado, USA
Anti-Osteonectin (mouse)	OST1	MU387-UC	1:400	Pressure cooker	BioGenex Laboratories	Fremont, California, USA
Anti-Osteopontin (mouse)	AKm2A1	sc-21741	1:300	Steamer pH 9	Santa Cruz Biotechnology, Inc.	Dallas, Texas, USA
Anti-tartrate resistant acid phosphatase (TRAP)(mouse)	9C5	EUL001	1:3000	Steamer pH 8	Kerafast, Inc.	Boston, Massachusetts, USA
Anti-runt-related transcription factor 2 (RUNX2) (mouse)	27-K	sc101145	1:50	Steamer pH 9	Santa Cruz Biotechnology, Inc.	Dallas, Texas, USA
Anti-CD68(mouse)	PG-M1	M0876	1:100	Steamer pH 6,1	Dako/Agilent Technologies, Inc.	Santa Clara, California, USA
Anti-CD163 (mouse)	10D6	NCL-L-CD163	1:50	Steamer pH 6,1	Leica Biosystems Newcastle Ltd.	Newcastle Upon Tyne, United Kingdom
Anti-Langerin (mouse)	12D6	392M-16	1:100	Pressure cooker	Cell Marque Tissue Diagnostics	Rocklin, California, USA

**Table 4:** Expression pattern of the analysed lesions for the studied antigens.

	Sample No.	HLA Class II	Cell Cycle				Bone Metabolism							Lineage		
		HLA-DR	Cyclin D1	Cyclin E	p16	p21	RANK	RANK-L	Osteoprotegerin	Osteonectin	Osteopontin	TRAP	RUNX2	CD68	CD163	Langerin
**Reactive lesions**																
Bony callus (*n* = 4)	1–4															
Sarcoidosis (*n* = 3)	5–7															
Sarcoid-like lesion (*n* = 2)	8–9															
Tuberculosis (*n* = 3)	10–12															
Foreign body granuloma (*n* = 3)	13–15															
Fibroid epulis (*n* = 2)	16–17															
Brown tumour (*n* = 2)	18a															
	18b															
**Neoplastic lesions**																
Giant cell tumour of bone (*n* = 11)	19–23															
	24a															
	24b															
	25a															
	25b															
	26a															
	26b															
Aneurysmal bone cyst (*n* = 7)	27–31															
	32a															
	32b															
Chondroblastoma (*n* = 3)	33–35															
Non-ossifying fibroma (*n* = 3)	36–38															
Tenosynovial giant cell tumour (*n* = 3)	39–41															
Xanthogranuloma (*n* = 1)	42															
	0%															
	up to 30%															
	30–70%															
	more than 70%															

**Table 5:** Studied molecules and their function.

Subgroup	Antigen	Short Description of Main Function
HLA class II	HLA-DR [25]	MHC ^1^ class II, expressed on antigen-presenting cells, B-cells, and T-cells, present exogenously derived antigens to T-lymphocytes.
Cell cycle	Cyclin D1 [26]	Forms a complex with CDK ^2^ 4 and 6, phosphorylates retinoblastoma protein, leading to cell cycle transition of G1- to S-phase.
	Cyclin E [27]	Forms a complex with CDK 2, allowing cell cycle transition from G1- to S-phase.
	p16 [28]	Inhibits cell cycle progression from G1- to S-phase, thereby slowing down cell division. The p16 gene constitutes the second most common tumour suppressor gene.
	p21 [29]	Universal CDK-inhibitor that inhibits CDK 2 and other CDKs, thus regulating the cell cycle progression in G1- and S-phase.
Bone Metabolism	RANK [30]	Receptor activator of nuclear factor κB, expressed in osteoclasts, takes part in their regulation, activated by RANK-L.
	RANK-L [31]	Receptor activator of nuclear factor κB ligand, released by osteoblast lineage cells, mediates osteoclastogenesis, activates mature osteoclasts and increases their survival time.
	Osteoprotegerin [32]	Secreted protein, decoy receptor of RANK-L, therefore blocking osteoclast production, also expressed on osteoblasts.
	Osteonectin [33]	Secreted glycoprotein, that binds calcium and mediates cell-matrix-interactions in bone and other tissues, is associated with tissue remodelling and mineralisation of collagen.
	Osteopontin [34]	Acidic extracellular matrix protein expressed in many different tissues. Is relevant for bone remodelling.
	TRAP [35]	Tartrate-resistant acid phosphatase, expressed in osteoclasts, activated macrophages, and dendritic cells. Biomarker for hairy cell leukaemia and increased bone metabolism. A natural substrate of TRAP is osteopontin.
	RUNX2 [36]	Master transcription factor that regulates cell differentiation into and proliferation of osteoblasts and chondrocytes.
Lineage	CD ^3^ 68 [37]	Membrane protein expressed on antigen-presenting cells like macrophages and dendritic cells
	CD163 [38]	Haemoglobin scavenger receptor present on resident macrophages and monocytes in normal and neoplastic conditions.
	Langerin [39]	CD 207, transmembrane protein expressed on Langerhans cells (immature dendritic cells) present in the epidermis and mucosa. Binds carbohydrates and pathogens.

^1^ MHC = major histocompatibility complex; ^2^ CDK = cyclin-dependent kinase; ^3^ CD = cluster of differentiation.

## Data Availability

The data presented in this study are available in this article.

## Supplementary Materials

The following supporting information can be downloaded at: https://www.mdpi.com/article/10.3390/cancers15143702/s1, Figure S1: HLA-DR-staining of sample 13 (foreign body granuloma), Figure S2: Cyclin E-staining of sample 6 (sarcoidosis), Figure S3: Cyclin E-staining of sample 14 (foreign body granuloma), Figure S4: RANK-staining of sample 1 (bony callus), and Figure S5: TRAP-staining of sample 24b (GC tumour of the bone). The bar marks the respective magnification.
