# Supplementary material for: Giant Cells of Various Lesions Are Characterised by Different Expression Patterns of HLA-Molecules and Molecules Involved in the Cell Cycle, Bone Metabolism, and Lineage Affiliation: An Immunohistochemical Study with a Review of the Literature
Source: Cancers (Basel). 2023 Jul 21;15(14):3702. doi: 10.3390/cancers15143702 (PMC10377796; doi:10.3390/cancers15143702)
Supplement: Supplementary file 1 [file cancers-15-03702-s001.zip › cancers-2446123-supplementary.pdf]

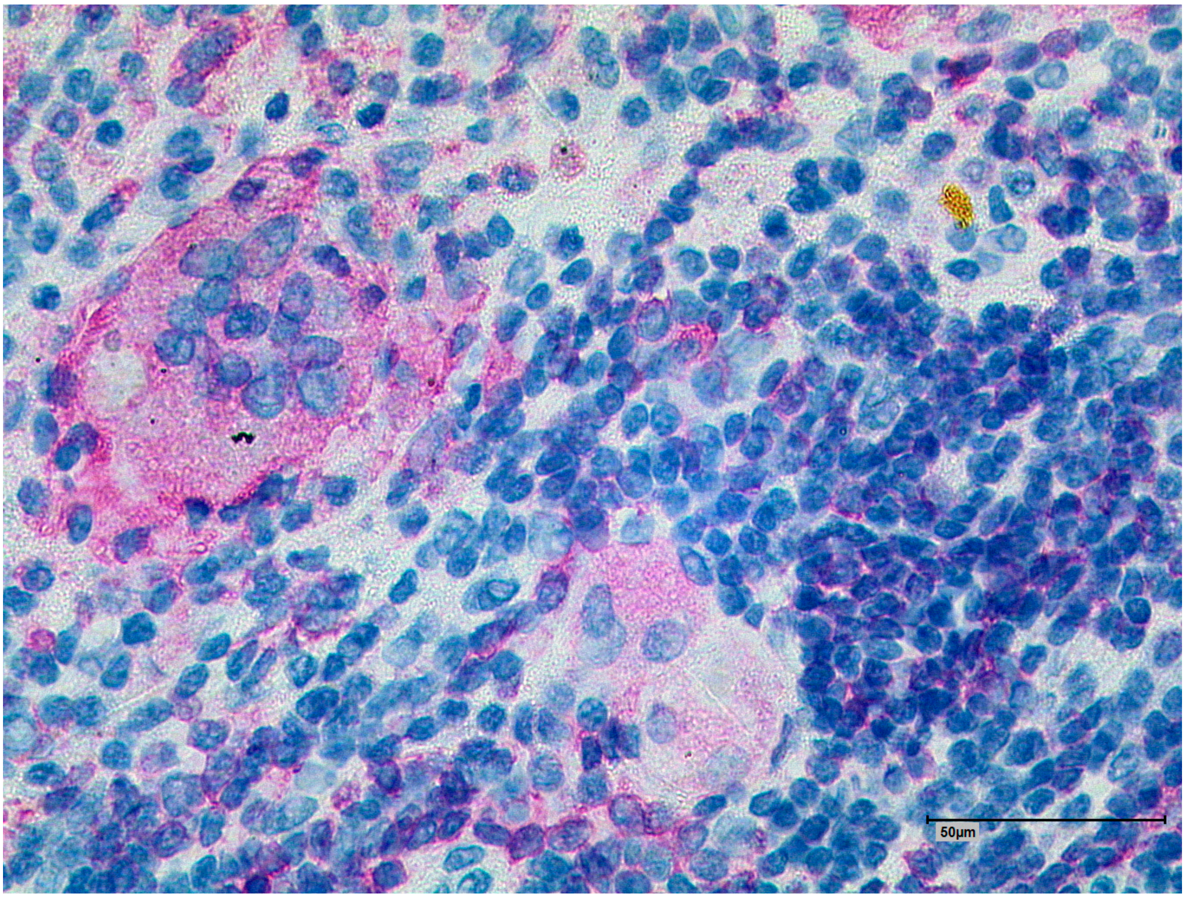

Figure S1: HLA-DR-staining of sample 13 (foreign body granuloma)

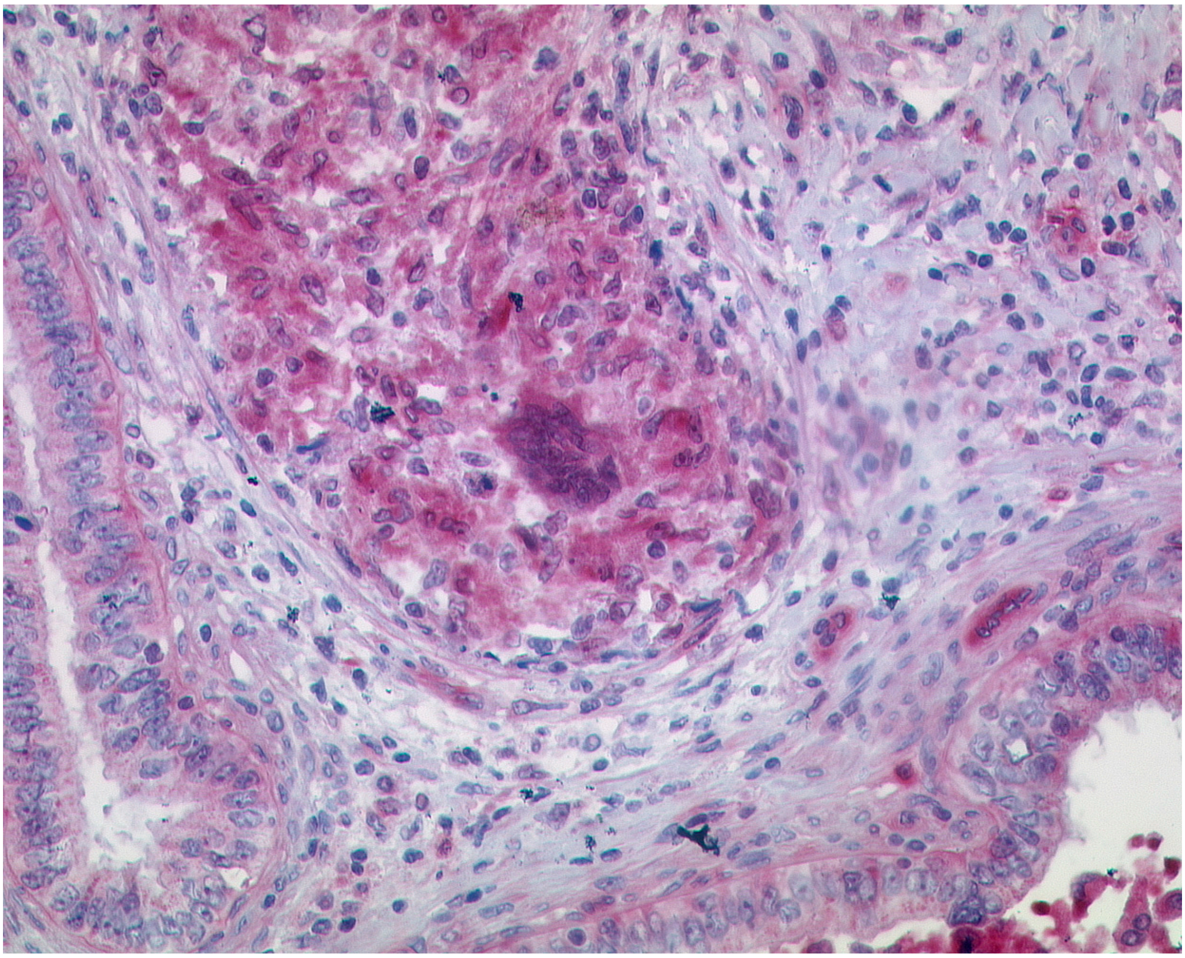
Figure S2: Cyclin E-staining of sample 6 (sarcoidosis)

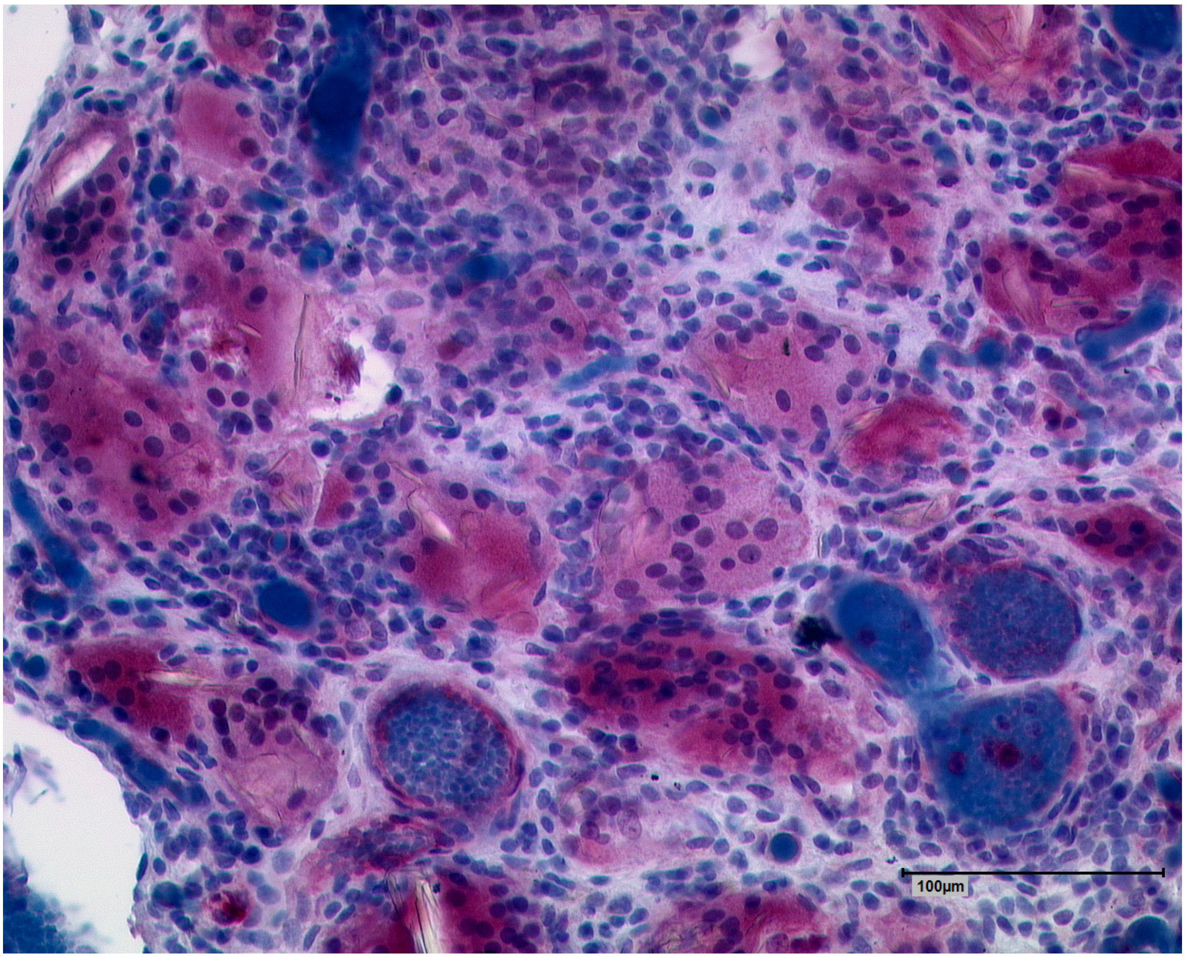

Figure S3: Cyclin E-staining of sample 14 (foreign body granuloma)

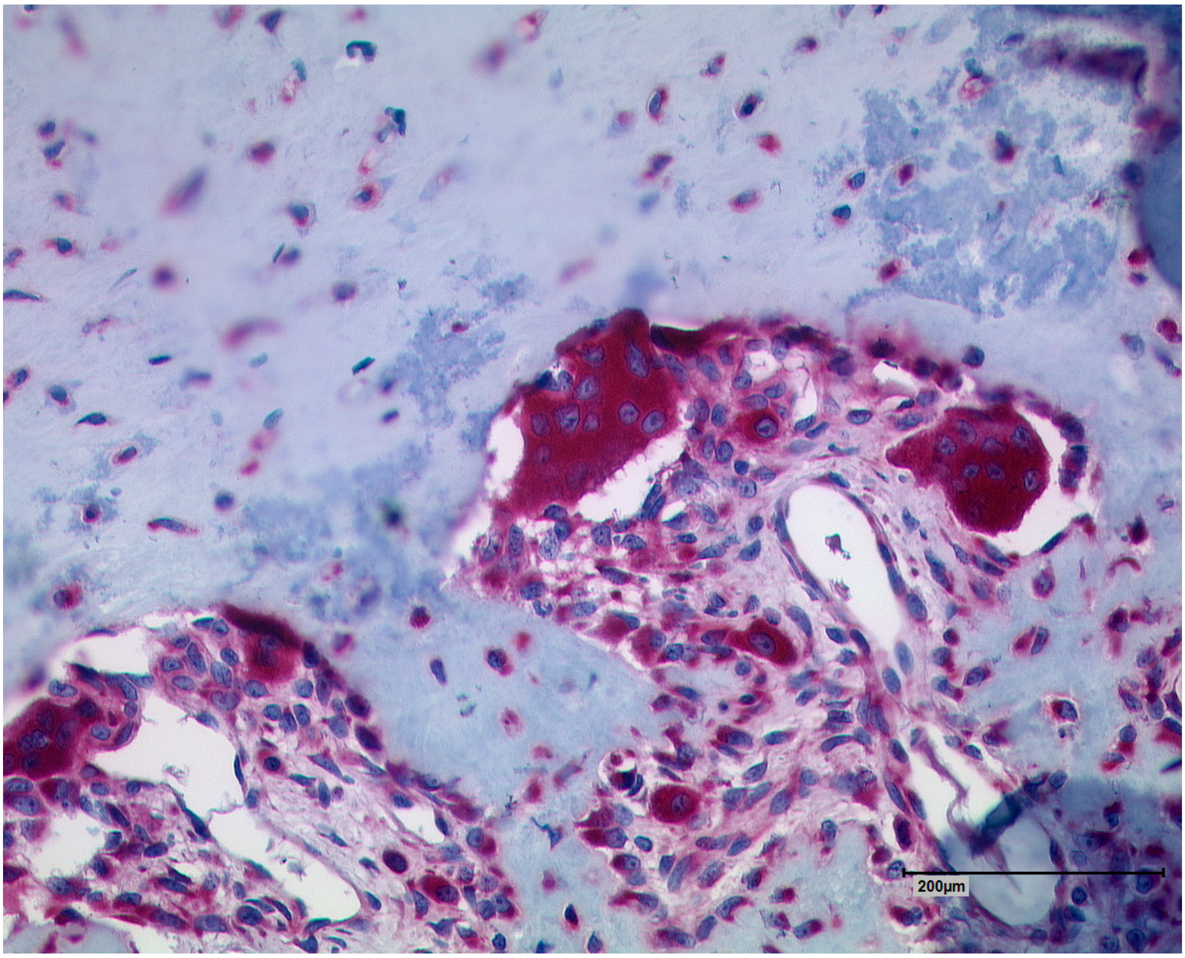

Figure S4: RANK-staining of sample 1 (bony callus)

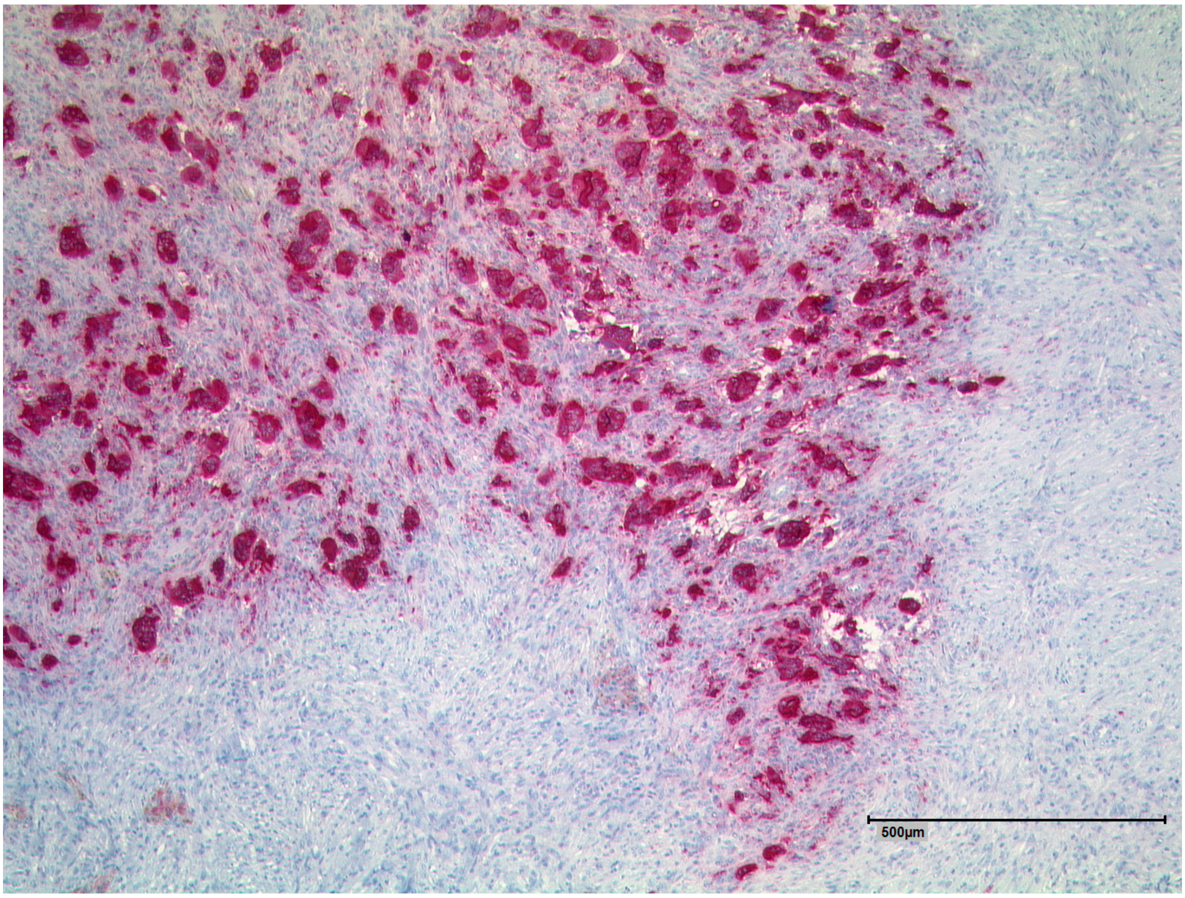

Figure S5: TRAP-staining of sample 24b (GC tumour of the bone).
